# A Review of Antibiotics, Antibiotic Resistant Bacteria, and Resistance Genes in Aquaculture: Occurrence, Contamination, and Transmission

**DOI:** 10.3390/toxics11050420

**Published:** 2023-04-30

**Authors:** Xia Yuan, Ziqing Lv, Zeyu Zhang, Yu Han, Zhiquan Liu, Hangjun Zhang

**Affiliations:** 1 School of Life and Environmental Sciences, Hangzhou Normal University, Hangzhou 311121, China; 2 Zhejiang Provincial Key Laboratory of Urban Wetlands and Regional Change, Hangzhou 311121, China; 3 School of Engineering, Hangzhou Normal University, Hangzhou 310018, China

**Keywords:** aquaculture, antibiotics, antibiotic resistant bacteria (ARB), antibiotic resistance genes (ARGs), contamination, transmission

## Abstract

Antibiotics are commonly used to prevent and control diseases in aquaculture. However, long-term/overuse of antibiotics not only leaves residues but results in the development of antibiotic resistant bacteria (ARB) and antibiotic resistance genes (ARGs). Antibiotics, ARB, and ARGs are widespread in aquaculture ecosystems. However, their impacts and interaction mechanisms in biotic and abiotic media remain to be clarified. In this paper, we summarized the detection methods, present status, and transfer mechanisms of antibiotics, ARB, and ARGs in water, sediment, and aquaculture organisms. Currently, the dominant methods of detecting antibiotics, ARB, and ARGs are UPLC−MS/MS, 16S rRNA sequencing, and metagenomics, respectively. Tetracyclines, macrolides, fluoroquinolones, and sulfonamides are most frequently detected in aquaculture. Generally, antibiotic concentrations and ARG abundance in sediment are much higher than those in water. Yet, no obvious patterns in the category of antibiotics or ARB are present in organisms or the environment. The key mechanisms of resistance to antibiotics in bacteria include reducing the cell membrane permeability, enhancing antibiotic efflux, and structural changes in antibiotic target proteins. Moreover, horizontal transfer is a major pathway for ARGs transfer, including conjugation, transformation, transduction, and vesiculation. Identifying, quantifying, and summarizing the interactions and transmission mechanisms of antibiotics, ARGs, and ARB would provide useful information for future disease diagnosis and scientific management in aquaculture.

## 1. Introduction

Aquaculture is one of the fastest growing industries in the world, helping solve food shortages and promote economic development. The Food and Agriculture Organization of the United Nations (FAO) predicted that the fish production of aquaculture could reach 109 million tons in 2030, with Asia as the dominant sector, accounting for 89% of total production [1]. Water quality in aquaculture is the key factor determining fish health. However, in practice, high-density breeding, pollution, or eutrophication of water bodies often lead to the decline of water quality and outbreaks of fish diseases [2]. Therefore, antibiotics have been used globally as aquaculture drugs or feed mixes to control fish diseases. On the one hand, antibiotics protect fish from infectious diseases to some extent [3]; however, on the other hand, the wide application or improper use of antibiotics creates problems of bacterial resistance, promoting the occurrence of antibiotic resistant bacteria (ARB) and antibiotic resistance genes (ARGs) in various aquatic environments.

Antibiotic pollution and the resulting contamination by ARB and ARGs have aroused wide concern [4]. ARGs contamination and their distribution in microbial populations throughout the biosphere is the result of continuous selection by the heavy anthropogenic use of antibiotics over the past few decades [5,6]. The high market demand for aquaculture products means that antibiotics are used in farmed products more than in humans, leading to increasing antibiotic resistance in pathogens [7]. As a new class of environmental pollutants, the spread of ARB and ARGs in aquaculture environmental media might be more harmful than antibiotics themselves [8]. ARGs and ARB could pose a threat to human health when ingested by humans via cultured products [9]. Moreover, prolonged abuse of antibiotics, as well as their recalcitrance, inevitably lead to large amounts of antibiotic residues in aquaculture environments. Antibiotic residues remaining in the environment could be further redistributed in water and sediments, having adverse effects on the ecological environment. Therefore, although many countries (including China, Japan, America, and the European Union) have banned the use of antibiotics, antibiotic contamination in aquaculture environments and production still exists. In addition, there is a very low probability that resistance could be selected and transferred by bacteria when the antibiotic selection pressure is high [10]. Overall, it is necessary to further detect and analyze the virulence and transmission mechanisms of antibiotic residues and the subsequent or emerging pollutants in aquaculture. Micro-organisms have developed efficient mechanisms that enable them to tolerate antibiotics, of which ARGs are one. ARGs are initially present in the genome of environmental bacteria or are produced by bacterial mutations [11] and can be transmitted by vertical gene transfer (VGT) [12] and horizontal gene transfer (HGT) [13]. HGT is the main route of ARG transmission, including conjugation, transformation, and transduction [14]. VGT involves the transfer of genetic material from the parent to the offspring [15]. Many studies have focused on antibiotics, ARB, and ARGs; however, an overview and determination of the mechanisms of antibiotic, ARB, and ARG interactions to date are also necessary.

This review carried out an integrated assessment of the threats to aquaculture associated with antibiotics, ARB, and ARGs in different media, including water, sediment, and organisms. We mainly examined the presence of major antibiotics, resistant bacteria, and ARGs in aquaculture water, sediment, and aquaculture organisms, exploring the connection and interaction mechanism of antibiotics, resistant bacteria, and ARGs in these three media, and provided a scientific basis for further in-depth research into the control of ARGs in the aquaculture environment in the future.

## 2. Methodology for the Study of Antibiotics, ARB, and ARGs

Understanding the variety, content, and abundance of antibiotics in the environment can help researchers precisely control and remove antibiotics [16]. To investigate the contamination status of antibiotics, multiple methods were designed to detect residual antibiotics in water, sediment, and aquaculture organisms. The main processes for analyzing antibiotics include extraction and detection. Currently, the commonly used detection methods are physicochemical, including liquid chromatography–tandem mass spectrometry (LC-MS/MS), high-performance liquid chromatography–tandem mass spectrometry (HPLC-MS/MS), and ultra-performance liquid chromatography–electrospray tandem mass spectrometry (UPLC-MS/MS). For example, nine antibiotics were detected using LC-MS/MS after filtering water samples through filter paper and using solid-phase extraction for sediment [17]. A study used solid-phase extraction to extract antibiotics and high-performance liquid chromatography–electrospray tandem mass spectrometry (HPLC-ESI-MS/MS) to analyze the concentrations of 10 antibiotics [18]. The recoveries were 83–117% for antibiotics in water and 52–105% for antibiotics in sediment, with relative standard deviations less than 10%. High-performance liquid chromatography–electrospray tandem mass spectrometry was also used to identify antibiotics in fish tissues, and the recoveries of the four antibiotics were 92.3%, 91.6%, 94.1%, and 93.7%, respectively [19]. All three methods have proved to be highly accurate and sensitive. Moreover, several other methods, such as microbial assays or immunochromatographic methods, have also been used to detect antibiotics. The microbial assay can qualitatively determine the antibiotic residues in a sample and has the advantage of being easy to perform; however, the detection time is long, and the method is not quantitative [20]. Tetracycline drug residues in Qinghai yak meat were assessed using a fluorescence immunochromatographic method, and the recovery ranged from 80 to 120%, indicating the high accuracy and sensitivity of the method [21]. However, one method can only detect one or one class of antibiotics, and the range of detection is extremely limited. In summary, for a highly efficient and accurate detection of antibiotics, UPLC-MS/MS or similar methods could be given priority in practical work and in research.

The detection of ARB includes the screening and identification of resistant strains. The initial screening of ARB is generally performed using drug sensitivity tests and passage culture [22]. Subsequently, 16S rRNA sequencing is performed on the cultured colonies to explore the taxonomy of the isolated strains [23]. Meanwhile, PCR amplification is performed to detect the ARGs. The drug sensitivity assay for ARB is also a phenotypic assay for ARGs [24]. Drug sensitivity assays include the paper slice method, the agar/broth dilution method, and the concentration gradient method, which require a highly pure culture, and the process is time consuming and laborious [25]. A relatively quicker method is to perform PCR amplification of the extracted DNA and 16S rRNA sequencing after purification. The abundance of operational taxonomic units (OTUs) is obtained from the sequencing results, and the abundance of bacteria carrying ARGs is also called the ARB abundance, which is verified through gene annotation [26].

The detection of ARGs allows clarification of their distribution characteristics and propagation patterns in the environment for follow-up research. There are numerous methods to study ARGs, such as fluorescent quantitative PCR, high-throughput fluorescent quantitative PCR, and macro-genomics. Most studies have used traditional fluorescence quantitative PCR. However, this method can only quantify a few or dozens of resistance genes [27]. The high-throughput fluorescence quantitative PCR technique is capable of quantifying hundreds of genes or multiple samples of ARGs in water; using high-throughput fluorescence quantitative PCR can identify 211 ARGs [28]. In recent years, many studies began to apply the metagenomics approach to detect ARGs, which is capable of detecting all ARGs in the environment and can help uncover novel resistance genes [29]. For instance, metagenomics has been used for detecting the abundance of ARGs in activated sludge and predicting ARG hosts [30] and screening multiple antibiotic resistant clones from soil non-pathogenic bacteria [31]. The above three methods have their own advantages, whereby one comprehensive method could be chosen according to a study’s requirements.

## 3. The Presence of Antibiotics, ARB, and ARGs in Aquaculture Environments

### 3.1. Type and Abundance of Antibiotics, ARB, and ARGs in Aquaculture Water

#### 3.1.1. Antibiotics in Aquaculture Water

At present, the level of antibiotics detected in the water bodies of aquaculture areas in China is generally at the level of ng L^−1^ to μg L^−1^. There are geographical differences in the level of antibiotics in farmed water because of the different farming habits and composition of farming species in different regions; however, the most commonly detected antibiotic types are basically the same. The commonly detected antibiotics in aquaculture water are tetracyclines, macrolides, fluoroquinolones, and sulfonamides (as shown in Table 1). The solubility, frequency, and usage of antibiotics are the main factors affecting antibiotic residues in water [32]. In general, antibiotic residues are also associated with the growth stages of cultured organisms, i.e., younger animals usually have less resistance and possibly need more antibiotics for disease control and growth promotion [33]. The concentration of antibiotics in water bodies is also influenced by environmental factors. For example, the average concentration of antibiotics in Zhuhai rivers and coastal waters was significantly higher in the dry season than in the rainy season [34]. This could be explained by the dilution effect of precipitation on antibiotics. Moreover, extreme temperatures could promote the biodegradation and photolysis of antibiotics [35], further reducing the level of antibiotic residues.

#### 3.1.2. Occurrence of ARB and ARGs in Aquaculture Water

The excessive use of antibiotics in aquaculture has resulted in the emergence or development of bacterial resistance [44]. For example, in water samples from tilapia farms, more than nine ARB species were detected, which were *Cyanobacteria* (26.5%), *Proteobacteria* (26.4%), *Actinobacteria* (11.0%), *Bacteroidetes* (9.8%), *Planctomycetes* (8.3%), *Verrucomicrobia* (6.1%), *Chloroflexi* (2.1%), *Chlorobi* (1.7%), *Firmicutes* (1.0%), and Other (4%). The ARB species in water varied from one location to another. The common bacterial groups in the water of Danjiangkou Reservoir were *Actinobacteria* (40.2%), *Proteobacteria* (21.1%), *Bacteroidetes* (17.7%), and *Cyanobacteria* (13.7%) [45]. Among them, the main types of ARGs were bacitracin (0.047 copies cell^−1^) and multidrug (0.016 copies cell^−1^) resistance genes [26]. Aspergillus, Trichoderma, and Bacteroidetes were the main phyla detected in a shrimp farm in Thailand [46]. Notably, the antibiotics used differed from one aquaculture area to another, and the above results indicated that bacteria usually have the ability to resist multiple antibiotics.

Bacterial resistance is associated with carried ARGs, which are commonly detected in the environment as an emerging contaminant [47]. To date, the research on ARGs in aquatic environments has mainly focused on sulfa drugs (e.g., trimethoprim), tetracycline, quinolones, and macrolides. The most frequently detected ARGs were sulfonamide ARGs, including sul1, sul2, and sul3; tetracycline ARGs, including tetA, tetB, tetC, tetD, tetE, tetG, tetH, tetM, tetO, tetQ, tetS, tetW, tetX, and tetZ; quinolone ARGs, including qnrA, qnrD, and qnrS; and macrolide ARGs, including ermC, ermA, emf (C), and mph (G) (Table 2). Their relative abundance in aquaculture waters ranges between 8.57 × 10^−7^ and 3.45 × 10^−2^ copies/16S rRNA (Table 2). In most studies, sulfa resistance genes and tetracycline resistance genes usually have high relative abundance in cultured waters. For example, a study detected the highest levels of sul2 in mariculture farms, with relative expression ranging from 0.029 to 0.075 [48]. Researchers detected abundant tetracycline resistance genes in fish culture environments in South Jeolla province and Jeju Island, among which tetB and tetD accounted for 74.8–98.0% of the total ARGs [49]. TetA, sul1, and sul2 were also frequently detected in aquaculture effluents [50].

### 3.2. Type and Abundance of Antibiotics, ARB, and ARGs in Aquaculture Sediments

#### 3.2.1. Occurrence of Antibiotics in Sediments

Sediment is a reservoir of antibiotics in cultured water bodies. Antibiotics in the water column can be adsorbed on suspended particulate matter and settle into the sediment [61]. In addition, antibiotics carried by unused bait entering natural water bodies would eventually enter the sediment [62]. Thus, although antibiotics are susceptible to photolysis and hydrolysis in water, the decay process of antibiotics is slowed down when they enter the sediment, eventually leading to long-term accumulation of antibiotics [40].

Currently, the most commonly mentioned categories of antibiotics in sediment are tetracyclines, sulfonamides, fluoroquinolones, and macrolides [63,64,65]. For example, tetracyclines and macrolide antibiotics were the main antibiotics detected in the sediments of the Liaohe River basin, with concentrations ranging from not detected (ND) to 405 ng g^−1^ and ND to 375 ng g^−1^, respectively. Fluoroquinolones and sulfonamides concentrations ranged from ND to 117 ng g^−1^ and ND to 2.63 ng g^−1^, respectively [66]. Fluoroquinolones were the main antibiotics in Lake Baiyangdian, accounting for 80.9% of the total concentration [67]. Similarly, fluoroquinolones accounted for the largest proportion of antibiotics detected in North Bay sediments, followed by sulfonamides, with these two compounds accounting for 91.0–100% of the total [43]. These studies also showed that the concentrations of antibiotics in sediments are generally 3~4 orders of magnitude higher than that in water, suggesting that sediments are a more stable site for the presence of antibiotics.

#### 3.2.2. Status of ARB and ARGs in Aquaculture Sediments

In aquaculture, antibiotic residues, feces, as well as feed with farmed organisms could gradually accumulate in the sediment, likely driving antibiotic-resistant pathogens to evolve [55,68]. Additionally, the dominant bacterial groups are commonly varied in different sedimentary environments. For example, sediments from shrimp culture ponds were studied for three culture years, and the five most abundant phyla were *Proteobacteria*, *Chloroflexi*, *Firmicutes*, *Bacteroidetes*, and *Planctomycetes* [69]. In the sediment of carp farm ponds, *Proteobacteria*, *Bacteroidetes*, and *Actinobacteria* were the dominant bacterial groups [70]. Moreover, in the sediment of a cultured pond during the suspension of bait [71], *Proteobacteria* were the dominant phylum, followed by *Chloroflexi*, *Bacteroidetes*, and *Cyanobacteria*. Potentially, different bacterial community structures in sediments with antibiotic residues could further induce ARGs contamination.

The types of ARGs in sediment were similar to those in the water body: sulfonamide ARGs, mainly including sul1, sul2, and sul3; tetracycline ARGs, including tetA, tetB, tetC, tetG, tetH, tetM, tetO, tetT, tetW, and tetX; quinolone ARGs, such as qnrA, qnrB, qnrD, and qnrS; and macrolide ARGs ermB and ermA (Table 2). The species of ARGs are different in different culture areas. The abundance of sulfonamide *sul1* and *sul2* in aquaculture sediments of the Pearl River Estuary was relatively high, with *sul1* considered a potential indicator of ARG [59]. Multidrug resistance genes were the most common ARGs in sediments from the Shatian Lake aquaculture area, with a detection rate of 24.5%. Fluoroquinolone ARGs represented 8.8%, tetracyclines ARGs represented 6.8%, and the detection rate of macrolides ARGs was 2.9% [63]. The relative abundance of ARGs in aquaculture environmental sediments was 1.25 × 10^−6^–1.08 × 10^−1^ copies/16S rRNA, which was higher than that in the water column. Yuan et al. [58] found that the spread of ARGs over time led to their accumulation in sediments, and sediments likely enhanced the spread of antibiotic resistance. The abundance of ARGs in sediments is also influenced by the culture pattern. It is shown that the abundance of ARGs in sediments from different modes of culture ponds (duck–fish mixed and separate culture), and the absolute abundance of ARGs, was significantly higher in integrated mode ponds than in non-integrated mode ponds [55]. Similarly, the abundance of ARGs in the sediment of an integrated duck and shrimp farm was more than one times higher than that of the shrimp monoculture ponds [72]. These findings implied that the abundance of ARGs was higher in the sediments of integrated mode ponds.

### 3.3. Type and Abundance of Antibiotics, ARB, and ARGs in Aquaculture Organisms

#### 3.3.1. Retention of Antibiotics in Aquaculture Organisms

Antibiotics are widely used to treat fish diseases and promote the output of aquatic products. However, intensive research has shown that only a small fraction of the antibiotics in aquaculture organisms could be broken down, with most of them being retained in the body of the aquaculture organism or excreted into the external environment. For example, a study found that only 20–30% of the antibiotics added are absorbed by fish, while most are input into water [73]. Taking the Haihe River as an example, the highest level of sulfamethoxazole in aquatic organisms was 68 ng g^−1^, and the average level of sulfamethoxazole in the water was 2.0 × 10^2^ ng L^−1^ [74]. The level of antibiotic residues in aquatic organisms is influenced by several factors. It is clear that the amount of antibiotics input artificially during the farming process is an important influencing factor. Secondly, antibiotics in the pristine environment might also affect antibiotic levels in aquatic organisms. Antibiotic residues were detected in wild marine organisms that had not been exposed to artificially added antibiotics, which was most likely caused by the uptake and enrichment of antibiotics in the pristine environment by aquatic animals [75]. In addition, fishmeal is also a potential factor for the continuous accumulation of antibiotics in aquatic animal products [76]. Approximately 25% of the harvest from global commercial marine fisheries is used for the production of fishmeal and fish oil. Fishmeal is produced from fish tissue and is often used as feed for aquaculture organisms [77]. About 30–70% of fish tissues are further processed and made into bait in China’s aquaculture industry [78], enriching the antibiotics in aquatic organisms from generation to generation, possibly resulting in greater levels of resistance [79]. Moreover, other pollutants (e.g., microplastics) could also aggravate the bioaccumulation of antibiotics in mollusks (such as *Mytilus* spp.).

The types of antibiotic residues in aquatic products are diverse. Intestinal antibiotics were detected in fish from five fish farms, revealing the presence of one sulfonamide, one quinolone, two tetracyclines, and three macrolides, among which the macrolide azithromycin was detected at a high rate in Tianjin, China [80]. Analysis of the intestines of South American white shrimp revealed the presence of sulfadiazine, ciprofloxacin, and norfloxacin in all samples, which belong to the sulfonamides and quinolones [81]. Moreover, antibiotics in the intestines of squid ginseng were detected, showing high levels of amikacin, gentamicin, ampicillin, and ribostamycin, which belong to the aminoglycoside and β-lactam antibiotic families [82]. Among them, the aminoglycoside amikacin was the most abundant antibiotic. Overall, in view of the abundance of diverse residual antibiotics in aquatic products, over 30 countries have banned the use of antibiotic growth promoters because of concerns over human health and environmental conservation.

#### 3.3.2. Status of ARB and ARGs in Aquaculture Organisms

The presence of ARB commonly varies among aquaculture organisms. In the intestinal flora of spotted catfish, the dominant phyla were *Bacteroidetes*, *Fusobacteria*, *Firmicutes* [83]. *Aeromonas hydrophila* and *Pseudomonas aeruginosa* were the most abundant bacteria in Nile tilapia [84]. The representative resistant bacteria in the intestines of South American shrimp were *Aeromonas*, *Kinetobacter*, *Escherichia*, *Sphingomonas*, *Enterobacter*, *Lactobacillus*, *Pantotrichum*, *Bacillus*, *Leuconostoc*, and *Vibrio* [85]. Differently, *Acinetobacter*, *Bacillus*, and *Psychrobacter* species were most prevalent in snow crab [86]. Thus, the dominant ARB would vary in different host organisms.

Exogenous addition of antibiotics in aquaculture would induce one or a variety of resistance genes produced by bacteria in aquaculture organisms, leading to antibiotic resistance development. In aquaculture organisms, there are four major classes of ARGs, comprising those conferring resistance to quinolones, macrolides, tetracyclines, and sulfonamides (Table 2), among which sul1, sul2, qnrA, qnrS, macB, tetA, and tetG were more likely to be detected [82,87]. Currently, metagenomics is increasingly used to detect ARGs in aquaculture organisms or an environmental medium, in addition to PCR assays or gene chip technology. Moreover, studies on ARGs in aquaculture organisms have mainly focused on intestinal flora because it is the dominant reservoir of ARGs, although other studies have focused on liver or hepatopancreas micro-organisms. The type and abundance of resistance genes in aquaculture organisms are also related to the commonly used local antibiotics. In areas where the use of sulfonamides is more prevalent than tetracyclines, sulfonamide resistance genes were prevalent and were present in the highest concentrations [88]. Notably, antibiotic misuse promotes the prevalence of ARGs, contributes to the formation of multidrug resistant bacteria, and poses a significant threat to human health and the environment [87].

## 4. Association between Antibiotics, ARB, and ARGs in Aquaculture Ecosystems

### 4.1. Mechanism of Interaction between Antibiotics, ARB, and ARGs

**Figure 1:**
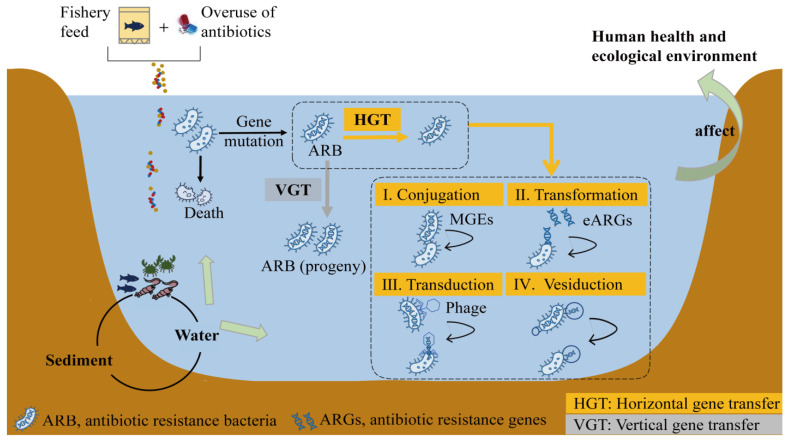
ARGs and ARB occurrence and transfer patterns in the aquaculture environment. MGE, mobile genetic element; eARGs, extracellular ARGs.

Generally, antibiotics act as bactericides by blocking the synthesis of bacterial cell membranes or cell walls to kill or inhibit pathogenic bacteria [89]. Research has shown that quinolone antibiotics inhibit bacterial growth by inhibiting bacterial DNA topoisomerase [90]. Macrolides bind to large ribosome subunits near peptidyl transferase centers and cause cell growth to stall by inhibiting protein synthesis [91]. Tetracyclines inhibit protein synthesis by preventing the attachment of aminoacyl-tRNA to the ribosomal receptor (A) site, thus exerting an antibacterial effect [92]. Sulfonamides interact with dihydrofolate synthase and prevent the formation of tetrahydrofolate. Tetrahydrofolic acid is a precursor of folic acid. Bacteria cannot grow and reproduce properly without folic acid [93]. Some pathogens could be killed quickly under antibiotic stress; yet, some also survive via corresponding resistance mechanisms related to protein or gene changes (Figure 1). There are two main mechanisms of antibiotic resistance in bacteria. One mechanism involves physically preventing the antibiotic from binding to the target protein, and the other involves altering the structure of the target protein on which the antibiotic acts to resist the antibiotic. The first mechanism includes reducing the permeability of the cell membrane to antibiotics and export of the antibiotic via efflux pumps. The second mechanism causes mutation of the target structure or temporary modification of the target structure [94].

The acquisition or spreading of ARGs in resistant bacteria occurs mainly through gene mutation and horizontal and vertical transfer (Figure 1). Residues of antibiotics in an aquaculture environment can exert antibiotic selection pressure on bacterial communities. Under this pressure, bacteria could acquire antibiotic resistance (via ARGs) through random chromosomal mutations [53]. ARGs are transmitted by two main processes: VGT, attributed to bacterial host propagation, and HGT, attributed to the transfer of ARGs between different bacterial cells through mobile elements [95]. HGT is an important pathway for the spread of ARGs, where mobile genetic elements (MGEs) transfer ARGs to other host bacteria, whether they are in intercellular contact or not [96]. Currently, the horizontal transfer includes four pathways: classical conjugation, transformation, transduction, and vesiduction [97]. Conjugation is the process of transferring ARGs from donor to recipient cells through direct cellular contact of MGEs, which can carry multiple ARGs [98]. Transformation is the uptake of extracellular plasmid or chromosomal DNA by competent cells and its stable integration into the bacterial genome to functionally express antibiotic resistance [99]. In addition, adsorbed extracellular ARGs (eARGs) tend to transfer to competent cells more than free eARGs [100]. Transduction is achieved by phage infection of donor cells. The DNA containing ARGs in the donor cell is encapsulated in the capsid of the phage. When the donor cell is lysed by the phage, the ARGs are transferred to the recipient cell along with phage infection. Vesiduction is a recently discovered mode of horizontal transfer, which refers to the spread of ARGs through extracellular vesicles. The membrane vesicles produced by the cells are able to carry the DNA of the source bacterial strain through the environment and protect the DNA from degradation [101].

In most cases, gene mutation, horizontal transfer, and vertical transfer work together at the same time. Briefly, the resistance to an antibiotic obtained by mutation of an antibiotic target gene can be transferred by a plasmid or phage. MGEs carrying ARGs are able to insert into bacterial chromosomes, resulting in chromosomally encoded resistance, which in turn allows such bacteria to transmit ARGs by vertical transfer. Some MGEs provide integrase genes that enable ARGs to bind to the host chromosome, while some MGEs are able to utilize integrase genes located on the host bacterial chromosome that allow them to excise themselves from the chromosome after they have completed their role [102]. After binding to the host chromosome, ARGs are selectively retained only when they confer an advantage on the host strain [103].

### 4.2. Ecological Risks of Antibiotics Residues, ARB, and ARGs in Aquatic Ecosystems

Early studies reported the adverse environmental effects of antibiotic residues in aquaculture environments, and subsequently, many studies have focused on the risk assessment of antibiotics in aquaculture environments [104]. The ecological risks are assessed using Formula (1) [105].

RQs = PEC or MEC/PNEC(1)

RQs, the risk quotients;
PEC, the predicted environmental concentration;
MEC, measured environmental concentration;
PNEC, predicted no-effect concentration.

The PNEC is computed using Formula (2).

PNECs = LC_50_ or EC_50_/AF (2)

LC_50_, half lethal concentration;
EC_50_, half maximal effective concentration;
AF, assessment factor.

The classification method of the RQ proposed by Norton et al. [106] can evaluate whether drugs pose risks to the ecosystem. If RQ > 1, the regional pollution will be considered to be in the high-risk state [107]; if 0.1 < RQ < 1, the pollution in this region is in a state of medium risk [108]; and if RQ < 0.1, the region is at a low risk of contamination [109]. Ashbolt et al. [110] proposed a threshold assessment method for the occurrence of ARB based on the minimum selective concentration (MSC). The pathogenic risk of a pathogen was assessed based on problem formulation (hazard, risk setting, and pathway), hazard exposure assessment (ARB and ARGs), and the dose–response relationship. This microbiological risk assessment (MRA) is used to assess the level of pathogen exposure and its subsequent risk to human health qualitatively or quantitatively. However, in vitro databases and computational toxicology studies are key factors for minimizing the uncertainty of ARG risk assessment. Compared with that for antibiotics, the overall assessment system for emerging contaminants is not very robust and needs further study.

## 5. Conclusions and Prospects

The detection method, concurrence, and contamination status of antibiotics, ARB, and ARGs in aquaculture ecosystems were summarized. Currently, the major methods for detecting antibiotics, ARB, and ARGs are UPLC-MS/MS, 16S rRNA sequencing, and metagenomics, respectively. Antibiotic concentrations in sediment are generally 3 to 4 orders of magnitude higher than those in water. Similarly, the relative abundance of ARGs in sediment is also much higher than that in aquaculture water. However, in aquaculture organisms, no obvious patterns in changes in the category or concentration of antibiotics were detected. There were also no clear patterns in the categories of ARB in the aquaculture environment. ARB in aquaculture mainly belonged to the *Cyanobacteria*, *Proteobacteria*, *Actinobacteria*, *Chloroflexi*, *Bacteroidetes*, and *Planctomycetes*. The interactions between antibiotics, ARB, and ARGs were also clarified. The key mechanisms of resistance to antibiotics in bacteria include reducing the permeability of the cell membrane to antibiotics, enhancing antibiotic efflux, and changing the structure of antibiotic target proteins. Furthermore, genetic mutations, horizontal transfer, and vertical transfer are the common methods of inducing and spreading ARGs. Horizontal transfer is an important pathway for the spread of ARGs, which includes conjugation, transformation, transduction, and vesiduction.

Based on the problems in current research works on ARGs, the following research questions and future outlook should be taken into consideration in future research studies: (1) Our current understanding of multidrug resistant ARGs is still relatively shallow, and the relevant databases need to be enriched; (2) eARGs contribute to the spread of antibiotic resistance in the environment, and their persistence is detrimental to the environment; however, this persistence in the environment is more complicated, still requiring further in-depth study; (3) the antibiotic resistome is extremely important for the study of ARG pollution control. Moreover, it is necessary to monitor antibiotics regularly to determine whether silent genes are expressed, such that corresponding measures can be taken for timely control of pollution.

## Figures and Tables

**Table 1:** The average amount of antibiotics detected in the aquaculture environment.

Antibiotics		Water	Sediment	Origin Location
Major Categories	Category	Concentration (ng·L^−1^) (Average/Range)	Concentration (µg·kg^−1^) (Average/Range)	
Tetracyclines	Oxytetracycline (OTC)	10.69	0.56	An aquaculture farm in Dongli District, Tianjin [36]
	Tetracycline (TC)	ND	ND~4.3	Trout farms along the Nera River [37]
	Chlortetracycline (CTC)	ND~50.32	-	Surface water of East Dongting Lake [38]
Macrolides	Roxithromycin (ROM)	1.22~110.66	-	Wusongkou, Yangtze River basin [38]
	Erythromycin (ERM)	ND	ND	Three forks of the Yangtze River basin [38]
Quinolones	Ciprofloxacin (CIP)	ND~2	ND~32.8	Trout farms along the Nera River [37]
	Norfloxacin (NOR)	ND~75.1	-	A trout aquaculture system located in the north of Portugal [39]
	Enrofloxacin (ENR)	0.5 × 10^3^	45.4	Tha Chin River in Thailand [40]
Sulfonamides	Sulfadiazine (SDZ)	ND~571	3–553	Trout farms along the Nera River [37]
	Sulfadimethoxine (SDM)	0.14 × 10^3^~ 0.88 × 10^3^	7.7	Aquaculture systems of northwestern Germany [41]
	Sulfamethoxazole (SMX)	0.04 × 10^6^~ 2.39 × 10^6^	4.77 × 10^3^~ 820.49 × 10^3^	Tiger shrimp farm in mangrove area of Vietnam [42]
	Methicillin (TMP)	0.93	0.89	Beibu Gulf marine farm [43]

Note: - represents data not explicitly reported.

**Table 2:** ARGs abundance in aquaculture water.

Antibiotics	ARGs	Abundance	Origin Location
Tetracycline	tetB/tetD/tetE/tetH/tetX/tetZ/tetQ	4.24 × 10^−3^~1.46 × 10^−2^ a	Fish culture environments in South Jeolla province and Jeju Island [49]
	tetA/tetC/tetG/tetM	-/-/-/1.16 × 10^2^ b	Hainan Dongzhai Port [51]
	tetA/tetB/tetC/tetH/tetM/tetO	5.46 × 10^−4^~1.61×10^−3^ a/2.05 × 10^−5^~6.35 × 10^−5^ a/4.71 × 10^−4^~1.68 × 10^−2^ a/8.47 × 10^−6^~2.37 × 10^−5^ a/8.84 × 10^−5^~4.97 × 10^−3^ a/5.90 × 10^−6^~6.99 × 10^−5^ a	West Coast of Pearl River Estuary [52]
	tetW/tetG/tetX	-	Jiangsu Province Baima Lake Aquaculture Farm [53]
	tetA/tetB/tetM/tetS/	-	Aquaculture farms in Sri Lanka [54]
Macrolides	ermC	-	Coastal farms in Jeollanam-do Province and Jeju Island, Korea [49]
	ermA	-	Pearl River Delta South China Zhongshan [55]
	emf(C)/mph(G)	-	A fish farm in Japan [56]
Quinolones	qnrA/qnrD/qnrS	-	Shuidongwan, Maoming City, Guangdong [57]
	qnrS	(100%); 9.97 × 10^−3^ a	Hangzhou Bay, Xiaoshan, Shaoxing, Cixi, Pinghu [58]
	qnrS	(100%); 8.57 × 10^−7^~3.45 × 10^−2^ a; 8.68~1.37 × 10^6^ b	Hainan Dongzhai Port [51]
	qnrA/qnrD/qnrS	-	Guangzhou Pearl River Delta Estuarine Aquaculture Zone [59]
Sulfonamides	sul1	3.29 × 10^2^ ± 4.81 × 10^2^ b; 2.72 × 10^2^ ± 3.57 × 10^2^ b; 4.08 × 10^2^ ± 2.06 × 10^2^ b	Waste lagoon of JFRC of the Ministry of Agriculture in Saudi Arabia [60]
	sul1/sul2/sul3	(100%/100%/25%); 1.94 × 10^−2^ a/-/-	Hangzhou Bay, Xiaoshan, Shaoxing, Cixi, Pinghu [58]
	sull/sul2	1.21 × 10^5^ b/5.13 × 10^5^ b	Hainan Dongzhai Port [51]

Note: a is relative abundance (copies/16S rRNA); b is absolute abundance (copies mL^−1^; copies g^−1^); - is data not explicitly reported; the detection rate is shown in brackets.

## Data Availability

Not applicable.
